# Adipic acid tolerance screening for potential adipic acid production hosts

**DOI:** 10.1186/s12934-017-0636-6

**Published:** 2017-02-01

**Authors:** Emma Karlsson, Valeria Mapelli, Lisbeth Olsson

**Affiliations:** 1 Department of Biology and Biological Engineering, Division of Industrial Biotechnology, Chalmers University of Technology, Gothenburg, Sweden; 2 Sacco S.r.l., Cadorago, CO Italy

**Keywords:** Adipic acid, Tolerance, Screening

## Abstract

**Background:**

Biobased processes for the production of adipic acid are of great interest to replace the current environmentally detrimental petrochemical production route. No efficient natural producer of adipic acid has yet been identified, but several approaches for pathway engineering have been established. Research has demonstrated that the microbial production of adipic acid is possible, but the yields and titres achieved so far are inadequate for commercialisation. A plausible explanation may be intolerance to adipic acid. Therefore, in this study, selected microorganisms, including yeasts, filamentous fungi and bacteria, typically used in microbial cell factories were considered to evaluate their tolerance to adipic acid.

**Results:**

Screening of yeasts and bacteria for tolerance to adipic acid was performed in microtitre plates, and in agar plates for *A. niger* in the presence of adipic acid over a broad range of concentration (0–684 mM). As the different dissociation state(s) of adipic acid may influence cells differently, cultivations were performed with at least two pH values. Yeasts and *A. niger* were found to tolerate substantially higher concentrations of adipic acid than bacteria, and were less affected by the undissociated form of adipic acid than bacteria. The yeast exhibiting the highest tolerance to adipic acid was *Candida viswanathii*, showing a reduction in maximum specific growth rate of no more than 10–15% at the highest concentration of adipic acid tested and the tolerance was not dependent on the dissociation state of the adipic acid.

**Conclusions:**

Tolerance to adipic acid was found to be substantially higher among yeasts and *A. niger* than bacteria. The explanation of the differences in adipic acid tolerance between the microorganisms investigated are likely related to fundamental differences in their physiology and metabolism. Among the yeasts investigated, *C. viswanathii* showed the highest tolerance and could be a potential host for a future microbial cell factory for adipic acid.

**Electronic supplementary material:**

The online version of this article (doi:10.1186/s12934-017-0636-6) contains supplementary material, which is available to authorized users.

## Background

Adipic acid is in great demand globally mainly for the production of nylon, although it is also used in the production of polyurethane, plasticizers, in controlled-release pharmaceuticals and as a flavouring and gelling aid in food. In recent years, biobased processes for the production of adipic acid have attracted considerable interest, as a sustainable alternative to the current, environmentally detrimental production process, which is based on petrochemical sources and chemical conversion [1–10]. In biobased processes, renewable raw materials can be converted into adipic acid with the aid of microorganisms. Microorganisms such as bacteria, yeasts, and filamentous fungi have long been used for the commercial production of various compounds with diverse applications, either by taking advantage of their natural metabolic properties, or by using genetic engineering to modify their metabolic routes to produce compounds of interest. The most common workhorses in microbial cell factories are the yeast *Saccharomyces cerevisiae* [11], the bacteria *Escherichia coli* and *Corynebacterium glutamicum* [12], and the filamentous fungus *Aspergillus niger* [13]. All these microorganisms are potential hosts for industrial adipic acid production. However, to achieve economic feasibility, the microorganisms should tolerate high titres of the acid, i.e., in the range 50–100 g L^−1^ [14, 15]. In addition, the microorganism should also preferably tolerate low pH, as the overall cost of processing at low pH is reduced, due to the lower amount of base required, and less complex downstream purification [16].

The dicarboxylic adipic acid may be present in three different forms depending on the pH of the environment and the p*K*_A_ values of the two carboxylic groups, namely undissociated; in which both carboxylic groups are protonated, semi-dissociated; in which only one of the two carboxylic groups is protonated, and dissociated; in which neither of the carboxylic groups is protonated (Table 1). The undissociated form of an acid can enter the cell via passive diffusion over the plasma membrane [17]. Once in the cytosol, the carboxylic groups become deprotonated due to the almost neutral pH in the cytosol, causing acid stress in the cell. The equilibrium between the three forms of adipic acid shifts towards undissociated as the environmental pH decreases (Table 1), and cells will thus experience higher stress with decreasing pH [18, 19] due to increased diffusion of undissociated adipic acid into the cell at a given total concentration of adipic acid. Although low pH is beneficial for downstream processing, this will probably increase the diffusion of undissociated adipic acid into the cell, causing increasing acid stress. Therefore, the pH of the process must be set so as to achieve a compromise between the requirements of downstream processing and stress to the cell. Cell stress may have several effects on cell physiology that could result in lower yield and/or productivity.

**Table 1:** Distribution of the forms of adipic acid at different pH values

Form	Structure	Amount (%)^a^			
		pH 4	pH 5	pH 6	pH 7
Undissociated		70.7	15.2	0.5	0.0
Semi-dissociated		28.2	60.6	20.0	2.5
Dissociated		1.1	24.1	79.5	97.5

^a^Amounts of the three forms of adipic acid was calculated using the Henderson–Hasselbalch equation: pH = p*K*a + log10([A^−^]/[HA]). The p*K*a values, p*K*a_1_ = 4.4 and p*K*a_2_ = 5.4, are from Pubchem

It has been demonstrated that the microbial production of adipic acid is possible [1–7, 20], but the yields and titres are too low for commercialisation. The reason for this could be linked to the specific metabolic pathway employed. However, the low yields and titres of microbially produced adipic acid so far could be due to acid stress and poor cellular tolerance to adipic acid itself. Product toxicity has been identified as one of the primary challenges in developing a bioprocess for organic acid production [15]. However, to the best of our knowledge, tolerance to adipic acid has not previously been addressed. Therefore, the aim of this study was to investigate which microorganism(s) have the potential for use in a microbial cell factory for the production of adipic acid, based on their tolerance to adipic acid. The growth of well-known microorganisms, including the bacteria *Escherichia coli* and *Corynebacterium glutamicum*, the yeasts *Saccharomyces cerevisiae, Zygosaccharomyces bailii* and *Candida viswanathii* and the filamentous fungus *Aspergillus niger*, was screened in the presence of adipic acid over a broad range of concentrations (0–684 mM). In addition, in order to investigate how/if different forms of adipic acid affect the microorganisms, cultures were performed at different environmental pH values.

## Methods

### Strains and cultivation media

Well-known microorganisms, were included in this study: the bacteria *Escherichia coli* K12 MG1655, *Corynebacterium glutamicum* [a wild-type strain (DSM 20300) and a lysine overproducing strain (ZW04)], the yeasts *Saccharomyces cerevisiae* [a lab strain (CEN.PK 113-7D) and an industrial strain (Ethanol Red)], *Zygosaccharomyces bailii* CBS 7555 and *Candida viswanathii* NCYC 997, and the filamentous fungi *Aspergillus niger* ATCC 1015 (see Table 2).

**Table 2:** Strains used in the present study

Species	Strain	References
*Escherichia coli*	K12 MG1655	[21]
*Corynebacterium glutamicum*	DSM 20300 (ATCC 13032)	[22]
*Corynebacterium glutamicum*	ZW04	[23]
*Saccharomyces cerevisiae*	CEN.PK 113-7D	[24]
*Saccharomyces cerevisiae*	Ethanol Red	
*Zygosaccharomyces bailii*	CBS 7555	
*Candida viswanathii*	NCYC 997 (ATCC 20336)	[25]
*Aspergillus niger*	ATCC 1015	[26]

Yeasts were cultivated in minimal medium [20 g L^−1^ glucose, 5 g L^−1^ (NH_4_)_2_SO_4_, 3 g L^−1^ KH_2_PO_4_, 1 g L^−1^ MgSO_4_·7H_2_O, 1 mL L^−1^ vitamin solution, 1 mL L^−1^ trace element solution]. Vitamin solution and trace element solution were prepared as previously described [27]. Potassium hydrogen phthalate buffer, 100 mM, was used to maintain the cultures at the desired pH. For the first and second pre-cultures pH 5.5 was used whereas the pH in the microplate was either pH 5 or pH 6. The *Corynebacterium glutamicum* strains were cultured in media reported in- and modified from [28]; specifically a complex medium (10 g L^−1^ tryptone, 5 g L^−1^ beef extract, 5 g L^−1^ yeast extract, 2.5 g L^−1^ NaCl, 10 g L^−1^ glucose and 5 g L^−1^ urea) or a minimal medium (10 g L^−1^ glucose, 1 g L^−1^ NaCl, 0.055 g L^−1^ CaCl_2_·2H_2_O, 0.2 g L^−1^ MgSO_4_·7H_2_O, 15 g L^−1^ (NH_4_)_2_SO_4_, 0.02 g L^−1^ FeSO_4_·7H_2_O, 0.0005 g L^−1^ biotin, 0.001 g L^−1^ thiamine hydrochloride, 0.03 g L^−1^ 3,4-dihydroxybenzoic acid, 10 mL L^−1^ of a 100 × trace element solution [29]). Potassium phosphate (100 mM), was used to buffer the medium at the desired pH. For the first and second pre-culture pH 7 was used whereas the pH in the microplate was either pH 7 or pH 6. *Escherichia coli* K12 was cultured in either LB medium or a modified M9 medium (4 g L^−1^ glucose, 0.241 g L^−1^ MgSO_4_, 0.011 g L^−1^ CaCl_2_, 1 g L^−1^ NH_4_Cl, 0.5 g L^−1^ NaCl). Potassium phosphate (100 mM), was used to buffer the culture medium at the desired pH. For the first and second pre-culture pH 7 was used whereas the pH in the microplate was either pH 7 or pH 6. *Aspergillus niger* was grown on agar plates containing minimal medium prepared according to [30] and 10 g L^−1^ glucose and 1.5–2% (*w*/*v*) agar. The pH of the minimal medium with agar was adjusted to desired pH (pH 4, pH 5 or pH 6) with addition of HCl or NaOH. To evaluate if addition of buffer would be needed in the solid media to prevent acidification during *A. niger* growth and organic acid production, pH indicators were added to the media; bromophenol blue (pH 4) and methyl red (pH 5 and pH 6) with final concentrations of 0.01% (*w*/*v*) or 0.005% (*w*/*v*). No detectable change in the pH of the solid medium was observed after 11 days of growth evaluation of *A. niger* on plates in the presence of the pH indicator bromophenol blue (data not shown). Since the growth of *A. niger* did not affect the pH of the agar-based medium, it was assumed that the dissociation state of adipic acid was stable over time.

### Medium supplements

Stock solutions of adipic acid were prepared and adjusted to the desired pH at 30 °C, using NaOH for stocks used for *A. niger* and KOH for stocks used in the microplate. For osmotic control cultivations KCl was added to the medium at concentrations giving the same osmolality as the adipic acid supplemented media. The osmolality of the KCl and adipic acid stock solutions was determined from the mean of three or four measurements and confirmed to have the same osmolality using a Fiske Micro-osmometer, model 210. For *A. niger* the osmotic control cultivations were not evaluated since growth on the surface of the agar plate was considered not to be affected by the different medium osmolality. All the stock solutions used were sterile filtered through a 0.2 µm aPES membrane (Thermo Fisher Scientific, Waltham, MA, USA). Adipic acid concentrations used for yeast and bacteria cultivations were: 0, 6, 12, 24, 48, 96, 192, 384 and 650 mM. Yeast cultures were buffered at pH 5 and pH 6; while bacteria cultures were buffered at pH 6 and pH 7. Adipic acid concentrations used in *A. niger* cultures on agar plates were: 0, 68, 171, 342, 513 and 684 mM at pH 6. The highest adipic acid concentration used for pH 5 was 513 mM, due to insolubility of 684 mM adipic acid at pH 5. The adipic acid concentrations in pH 4 medium were: 0, 34, 68, 103 and 137 mM.

### Yeast and bacterial inocula

The first pre-culture was prepared by inoculating a single colony in 10 mL of medium in a 100 mL Erlenmeyer shake flask (E-flask) and grown overnight. The first pre-culture was used as the inoculum for the second pre-culture in 25 mL medium in a 250 mL E-flask with initial optical density (OD_600_). Exponentially growing cells were harvested by centrifugation (3000×*g*, 3 min, room temperature). The supernatant was discarded and the cell pellet was re-suspended in fresh medium; the same medium as used in the following microplate cultivation. This culture was used as the inoculum for the microplate, inoculated at initial OD_600_ of 0.2. The media and growth conditions for each microorganism are summarized in Table 3.

**Table 3:** Media and growth conditions for yeasts and bacteria used in this study

Microorganism	Medium			Growth conditions E-flask	
	1st pre-culture	2nd pre-culture	Microplate	Temperature (°C)	Shaking (rpm)
*E. coli*	LB medium (pH 7)	Modified M9 medium (pH 7)	Modified M9 medium (pH 6 or 7)	37	200
*C. clutamicum*	Complex medium (pH 7)	Minimal medium (pH 7)	Minimal medium (pH 6 or 7)	30	200
Yeasts	Minimal medium (pH 5.5)	Minimal medium (pH 5)	Minimal medium (pH 5 or 6)	30	180

### Evaluation of adipic acid tolerance in microtiter plates cultures

The cell growth kinetics of yeasts and bacteria was monitored in 145 µL aerobic microscale cultures at 30 °C using Bioscreen C MBR equipment (Oy Growth Curves Ab Ltd, Finland). For each set of experimental conditions a minimum of 5 replicates were used. The cell cultures were shaken continuously at the settings “high amplitude” and “fast speed” and stopped 5 s prior each optical reading. The cell density was measured optically every 15–20 min using a wideband 450–580 nm wavelength filter. The cell density values given by the Bioscreen were converted to equivalent OD_600_ using Eq. (1).

1 $$OD_{600} = \frac{{OD_{bioscreen} }}{{Pathlength\,({\text{cm}}) \times 1.32}}$$

The non-linear correlation between optical density and cell density at high cell densities was corrected using Eq. (2) [31].

2 $$OD_{corrected} = OD_{observed} + 0.449 \times OD_{observed}^{2} + 0.191 \times OD_{observed}^{3}$$

The maximum specific growth rate (µ_max_) was calculated, together with values of the coefficient of determination (R^2^), from the steepest part of the ln(OD_corrected_) curve.

The relative µ_max_, corrected for the effect of osmotic pressure (Rel.µ_max,osm.cor_), was calculated using Eq. (3).

3 $$Rel.\upmu_{max,osm.cor} = \frac{{\upmu_{max, control} -\upmu_{max, osmotic test} +\upmu_{max, adipic acid } }}{{\upmu_{max, control} }}$$

To test for differences between two samples, statistical analysis was performed using students *T* test, assuming two-tailed distributions, equal variance and the accepted risk level set to <5% (i.e. the p value <0.05).

### Spore solution preparation and growth measurements of *Aspergillus niger*

Black spores from a single colony were spread on a PDA plate and incubated at 30 °C for 3 days. Spores were harvested in 5 mL 0.02% (v/v) Tween solution, pH 6.9. The concentration of spores was determined with a Neubauer improved haemocytometer with 0.100 mm depth. For growth measurements, 2 µL of spore solution containing 500 spores µL^−1^ were added in triplicate to each plate. The pH of the plates were either pH 4, pH 5 or pH 6. All plates were incubated in 30 °C. The growth of *A. niger* was measured every 24 h as the diameter (mm) of the mycelium edges using a ruler.

### Estimation of the adipic acid concentration causing a certain reduction in µ_max_

Graphs of relative µ_max,osm.cor_ were plotted as a function of adipic acid concentration, and linear regression was performed, assuming a linear relationship. For adipic acid concentrations where no growth was observed, zero growth was defined from that concentration and above, assuming a linear trend. From these graphs, the adipic acid concentration causing a certain reduction in µ_max_ was estimated, facilitating comparison of the tolerance of the microorganisms to adipic acid.

### Evaluation of growth kinetics during respiratory growth phase

In the present study, when a second growth phase was observed following a diauxic shift and could be referred to growth under respiratory metabolism, the fold change in OD for this phase was calculated. A fold change <1, i.e., less than a doubling in OD, was not considered as real growth and no further analysis was performed. Throughout this paper, unless otherwise stated, when talking about growth it means growth with a fold change greater or equal to 1. During the time of the diauxic shift, no growth occurs and a lag phase may be observed. In this study the lag phase was defined and calculated as the time between the last time point included in the calculation of µ_max_ during respiro-fermentation and the first time point included in the calculation of µ_max_ during respiration.

## Results

The tolerance to adipic acid was evaluated for selected microorganisms including yeasts, bacteria and filamentous fungi. Yeasts and bacteria were grown in microscale (145 µL) cultures and the filamentous fungus *Aspergillus niger* on agar plates, all in the presence of different concentrations of adipic acid, ranging from 0 mM to 684 mM. Cultures were grown at the pH optimal for each microorganism, and at least one additional pH value to reveal any possible effects of the dissociation state of adipic acid on the tolerance of the microorganism.

### Comparison of the effects caused by adipic acid and osmotic pressure

A general trend was observed of a decrease in µ_max_ upon increasing the adipic acid concentration. The decrease in the specific growth rate was greater for cells grown in the presence of adipic acid than in those grown under the equivalent osmotic pressure caused by the addition of KCl.

Representative growth curves for CEN.PK 113.7D cells cultivated in adipic acid and KCL (osmotic controls) both at pH 5 are shown in Fig. 2. Growth curves for the other microorganisms are reported in the Additional file 1.

### Effect of adipic acid on bacteria

Three bacterial strains were included in this study: two strains of *Corynebacterium glutamicum*; the wild type DSM 20300 (ATCC 13032) and the genetically modified strain ZW04 deriving from DSM 20300 able to over-produce l-lysine [23], and the *Escherichia coli* strain K12 MG1655. All bacteria were cultivated at their optimal pH, that is pH 7, and also at pH 6.

#### *Corynebacterium glutamicum*

##### *Effect of adipic acid on the growth of C. glutamicum* DSM 20300 at pH 7

**Fig. 1:**
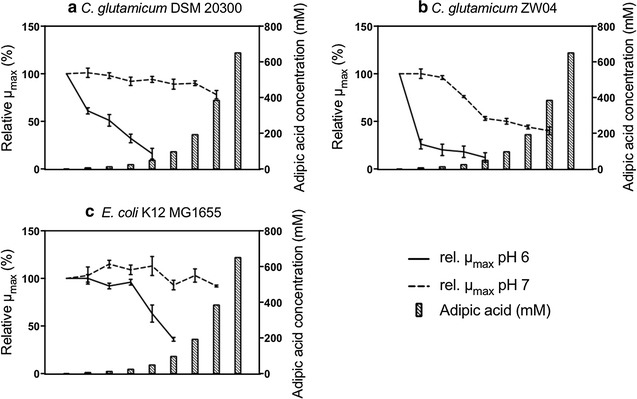
Effect of adipic acid on µ_max_ of *C. glutamicum* DSM 20300 (**a**), *C. glutamicum* ZW04 (**b**) and *E. coli* K12 MG1655 (**c**). The results are expressed as relative µ_max_, corrected for the effect of osmotic pressure, to the µ_max_ of cells grown without adipic acid. The results are given as the mean ± standard deviation from five replicates. The concentrations of adipic acid are: 0, 6, 12, 24, 48, 96, 192, 384 and 650 mM

*C. glutamicum* DSM 20300 was able to grow in the presence of all the adipic acid concentrations used, except 650 mM, at which growth was completely inhibited (Additional file 1: Fig. S1). There was a trend towards a slight decrease in µ_max_ with increasing adipic acid concentration, reaching a maximum of 22% (±4%) reduction in the presence of 384 mM adipic acid (Fig. 1a).

##### Effect of pH on C. glutamicum DSM 20300 grown in the presence of adipic acid

When the pH was decreased from 7 to 6, the cells lost their ability to grow at concentrations of adipic acid between 48 mM and 96 mM. Apart from tolerating lower titres of adipic acid when the pH was reduced, µ_max_ was greatly affected showing a clear decrease with increasing concentration of adipic acid. The maximum specific growth rate was reduced by 39% (±3%) already at 6 mM adipic acid, and at 48 mM it was reduced by 84% (±6%) (Fig. 1a). The final OD was also greatly affected when the pH was lowered to pH 6, and the final OD decreased with increasing adipic acid concentration, which was not caused by the osmotic pressure (Additional file 1: Fig. S1). At 48 mM adipic acid, the final OD was reduced by 59% (±1%) compared to the control with no adipic acid. The reduction in the final OD with increasing adipic acid concentration was negligible for cells grown at pH 7.

##### *Effect of adipic acid on the growth of C. glutamicum* ZW04 at pH 7

*C. glutamicum* ZW04 was able to grow at all concentrations of adipic acid tested except 650 mM, where no growth was observed (Additional file 1: Fig. S2). A considerable decrease in µ_max_ was observed between 12 mM and 48 mM, from 4% (±2%) to 47% (±2%). The maximum specific growth rate was further reduced with increasing adipic acid concentration, but the decline was not as large. The greatest reduction in µ_max_, 60% (±4%) was seen at 384 mM adipic acid (Fig. 1b). The final OD decreased with increasing adipic acid concentration, by 54% (±1%) at 384 mM adipic acid compared with the control with no adipic acid. The reduction in final OD in the osmotic controls was negligible (Additional file 1: Fig. S2).

##### Effect of pH on *C. glutamicum* ZW04 grown in the presence of adipic acid

When decreasing the pH from 7 to 6, the cells lost their ability to grow at concentrations of adipic acid between 48 mM and 96 mM. Apart from tolerating less high titres of adipic acid when the pH was reduced, µ_max_ was greatly affected: at 6 mM adipic acid µ_max_ was reduced by 74% (±5%), while at 48 mM adipic acid µ_max_ was reduced by 88% (±5%) (Fig. 1b). The final OD also decreased with increasing adipic acid concentration, and at 48 mM final OD was reduced by 72% (±1%) compared to control. In the osmotic control no reduction in final OD was evident up to osmotic pressures corresponding to 48 mM adipic acid. At higher osmotic pressures, a decrease in final OD was observed with increasing osmotic pressure (Additional file 1: Fig. S2).

#### *Escherichia coli*

##### Effect of adipic acid on the growth of *E. coli* K12 MG1655 at pH 7

*E. coli* K12 MG1655 was able to grow at all concentrations of adipic acid, except 650 mM. It is interesting to note that this strain was the only one among all yeasts and bacteria investigated that could not grow at an osmotic pressure equivalent to 650 mM of adipic acid. However, at 384 mM adipic acid µ_max_ was significantly more affected than in the osmotic control, showing that adipic acid affected growth rate more than osmotic pressure. There was no clear trend in µ_max_ as a function of increasing adipic acid, and in fact a slight increase in µ_max_ was observed between 12 mM and 48 mM of adipic acid. In cultures with 384 mM adipic acid, µ_max_ was only reduced by 8% (±1%) (Fig. 1c). It should also be noted that there were indications of a second growth phase up to 48 mM adipic acid, but the fold change in OD was well below 1 (Additional file 1: Fig. S3).

##### Effect of pH on E. coli K12 MG1655 grown in the presence of adipic acid

At pH 6, *E. coli* growth was completely inhibited at adipic acid concentrations between 96 mM and 192 mM. Apart from tolerating lower concentrations of adipic acid when the pH was reduced, µ_max_ was greatly affected between 24 mM and 96 mM adipic acid, being only 4% (±3%) at 24 mM but 64% (±2%) at 96 mM (Fig. 1c). A second growth phase was only observed for the control with no adipic acid. The final OD was greatly affected by increasing adipic acid concentration and at 96 mM adipic acid, the final maximum OD was reduced with 64% (±3%) compared to the control with no adipic acid after the first growth phase. The reduction in final OD in the osmotic controls, corresponding to adipic acid concentration of 96 mM, was negligible (Additional file 1: Fig. S3).

##### Comparison of the tolerance of bacteria to adipic acid

At pH 7, all bacteria were able to grow at 384 mM adipic acid. At 384 mM adipic acid, *E. coli* showed the least reduction in µ_max_, followed by *C. glutamicum* DSM 20300. The lysine-producing strain *C. glutamicum* ZW04 showed the least tolerance to adipic acid. When the pH was decreased to pH 6, the tolerance of all investigated bacteria species/strains to adipic acid decreased. Even at lower concentrations of adipic acid, µ_max_ was much more affected at pH 6 than at pH 7. Overall, *E. coli* K12 MG1655 was the bacterial strain that tolerated adipic acid best, whereas the lysine-producing strain *C. glutamicum* ZW04 was the least tolerant to adipic acid. At pH 6, the *C. glutamicum* strain ZW04 showed the same level of reduction in µ_max_ as the DSM 20300 strain at 48 mM adipic acid, but since the reduction in µ_max_ at lower adipic acid concentration, was greater for ZW04 strain than for the wild type (Fig. 1a, b) it can be deduced that the tolerance of *C. glutamicum* ZW04 to adipic acid is lower than that of the wild type strain, also at pH 6. In Table 4 the reduction in µ_max_ at the highest concentrations of adipic acid allowing growth is summarized for all bacteria at both pH 6 and pH 7.

**Table 4:** Reduction in µ_max_ for bacteria at the highest concentration of adipic acid allowing growth

Species	Strains	pH 7		pH 6	
		Reduction in µ_max_ (%)	Adipic acid (mM)	Reduction in µ_max_ (%)	Adipic acid (mM)
*C. glutamicum*	DSM 20300	22 ± 4	384	84 ± 6	48
*C. glutamicum*	ZW04	60 ± 4	384	88 ± 5	48
*E. coli*	K12 MG1655	8 ± 1	384	64 ± 2	96

The reduction in µ_max_ is corrected for osmotic pressure and compared to control with no adipic acid. Values given are the mean ± standard deviation of five replicates

##### Effect of adipic acid on yeasts

Three yeast species were investigated: two strains of *Saccharomyces cerevisiae* (the laboratory strain CEN.PK 113-7D and the industrial strain Ethanol Red) the food spoilage yeast *Zygosaccharomyces bailii* CBS 7555, known to tolerate high concentrations of various acids such as acetic acid [32], and *Candida viswanathii* NCYC 997 (ATCC 20336), a strain known to produce lipases (triacylglycerol acyl hydrolase, EC 3.1.1.3) allowing this yeast to grow on fatty acids and alkanes as sole carbon source [7, 33] and of high industrial interest for the production of adipic acid [34]. All yeasts were cultivated at their optimal pH, which is pH 5. In addition, yeasts were also cultivated at pH 6, yielding a lower concentration of undissociated acid compared to pH 5.

#### *Saccharomyces cerevisiae*

##### Effect of adipic acid on the growth of CEN.PK 113-7D at pH 5

**Fig. 2:**
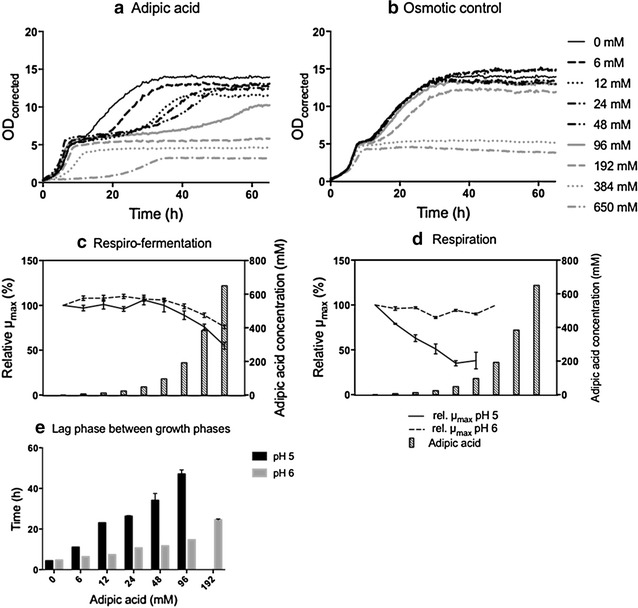
Effect of adipic acid on the growth kinetics of *S. cerevisiae* CEN.PK 113-7D. Growth curves for cultures at pH 5 in the presence of adipic acid (**a**) and osmotic pressure control experiments (**b**). The corresponding concentration of adipic acid is reported in the osmotic pressure experiments. Relative µ_max_ during respiro-fermentative growth (**c**) and respiratory growth (**d**). The results are expressed as relative µ_max_, corrected for the effect of osmotic pressure, to the µ_max_ of cells grown without adipic acid. The results are given as the mean ± standard deviation from five replicates. The concentrations of adipic acid are: 0, 6, 12, 24, 48, 96, 192, 384 and 650 mM. Lag phase between the respiro-fermentative and the respiratory growth phase (**e**) where the *bars* represent mean ± standard deviation for five replicates

Under the first growth phase, during respiro-fermentation, CEN.PK 113-7D cells were able to grow in the presence of all concentrations of adipic acid tested (Fig. 2a, b). An initial lag phase was observed at the highest acid concentration. Between adipic acid concentrations of 96 mM and 196 mM, µ_max_ was significantly reduced, and continued to decrease with increasing adipic acid concentration. At a concentration of 650 mM the reduction in µ_max_ was 45% (±4%) (Fig. 2c) and the final OD was 39% (±1%) lower compared to the control with no adipic acid. The reduction in the final OD was significantly greater than that caused by osmotic pressure, where the reduction in the final OD was 17% (±1%).

After the diauxic shift, when yeast metabolism changed from respiro-fermentative to fully respiratory, growth was observed up to an adipic acid concentration of 96 mM, while respiratory growth was completely inhibited at higher acid concentrations (Fig. 2a). The inhibition in respiratory growth observed between 96 mM and 192 mM adipic acid was not caused by the osmotic pressure, although osmotic pressure played a major role in inhibiting respiratory growth at concentrations of 384 and 650 mM. During respiratory growth, µ_max_ decreased significantly with increasing adipic acid concentration, compared to the control. The reduction in µ_max_ was 62% (±9%) at 96 mM adipic acid (Fig. 2d) and the final OD was reduced by 26% (±15%) compared to the control. The final OD of the osmotic control was not affected by applying an osmotic pressure equivalent to 96 mM adipic acid.

It was also observed that the lag phase between the respiro-fermentative and respiratory growth phases, increased with increasing adipic acid concentration. In the control with no adipic acid, the lag phase was 4.3 h while it increased to 47 h (±2 h) with 96 mM adipic acid (Fig. 2e). This increase in time was not observed in the osmotic control measurements (data not shown).

##### Effect of pH on CEN.PK 113-7D grown in the presence of adipic acid

When the pH of the medium was buffered to pH 6, the most important differences compared to pH 5 during respiro-fermentative growth were the lack of a lag phase in the presence of 650 mM adipic acid and a smaller decrease in µ_max_ with increasing adipic acid concentration. At 650 mM adipic acid, µ_max_ was reduced by 24% (±2%), which was significantly less than at pH 5, where the reduction in µ_max_ was 45% (±4%) (Fig. 2c). The final OD after the first growth phase was not considerably affected by the change in pH (Additional file 1: Fig. S4).

During respiratory growth, no decrease in µ_max_ was observed at pH 6 with increasing adipic acid concentration. Cells were able to grow at concentrations of adipic acid up to 192 mM, whereas at pH 5, respiratory growth was only observed up to 96 mM adipic acid (Fig. 2d). Furthermore, the lag phase between the respiro-fermentative and the respiratory growth phases was less affected at pH 6 than at pH 5 being 47 h (±2 h) with 96 mM adipic acid at pH 5, and 15 h at pH 6 (Fig. 2e). In addition, the maximum OD was less affected at increasing adipic acid concentrations at pH 6 than at pH 5. No significant effect was seen on µ_max_ in the osmotic control measurements at pH 6 up to 192 mM adipic acid, while at osmotic pressures corresponding to 384 mM and 650 mM adipic acid, osmotic stress played a considerable role, inhibiting µ_max_ and negatively affecting the final OD reached (Additional file 1: Fig. S7).

##### Effect of adipic acid on the growth of Ethanol Red at pH 5

**Fig. 3:**
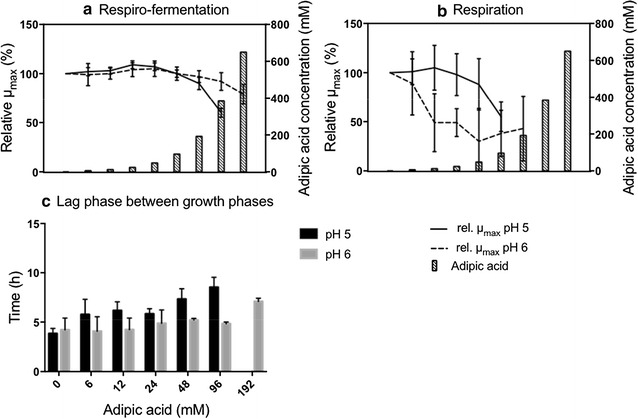
Effect of adipic acid on the growth kinetics of *S. cerevisiae* Ethanol Red. Relative µ_max_ during respiro-fermentative growth (**a**) and respiratory growth (**b**). Lag phase between the respiro-fermentative and the respiratory growth phase (**c**). The results in A and B are expressed as relative µ_max_, corrected for the effect of osmotic pressure, to the µ_max_ of cells grown without adipic acid. The concentrations of adipic acid are: 0, 6, 12, 24, 48, 96, 192, 384 and 650 mM. All results are given as the mean ± standard deviation from ten replicates

During respiro-fermentation, cells of Ethanol Red were able to grow in the presence of all concentrations of adipic acid tested, except 650 mM. µ_max_ showed a slight increase between 12 mM and 48 mM adipic acid, whereas at 192 mM it decreased significantly, compared to the control with no adipic acid, and continued to decrease with increasing adipic acid concentration. At 384 mM adipic acid, the reduction in µ_max_ was 40% (±5%) (Fig. 3a) and the final OD before the diauxic shift, in relation to control, with no adipic acid was reduced by 16% (±1%), which was not observed in the osmotic control (Additional file 1: Figs. S5, S6).

After the diauxic shift, a fold change of OD ≥ 1 was observed up to 96 mM adipic acid, but this was reduced to 0 at 192 mM adipic acid when the growth of Ethanol Red was inhibited. At 96 mM adipic acid, there was a significant reduction (45 ± 15%) in µ_max_ compared to the control with no adipic acid (Fig. 3b). The values of µ_max_ obtained during respiration showed large error bars, possibly indicating that Ethanol Red may be less efficient than the CEN.PK 113-7D strain in respiratory metabolism. This observation could be related to the fact that Ethanol Red has been evolved and selected for its highly fermentative metabolism and high ethanol yields. The maximum OD after the second growth phase was reduced by 20% (±12%) in the presence of 96 mM of adipic acid, and this reduction was not due to osmotic pressure (Additional file 1: Figs. S5, S6).

The lag phase between respiro-fermentative and respiratory growth phases increased slightly with increasing adipic acid concentration. In the control experiment with no adipic acid added the lag phase was 3.8 h (±0.5 h), and increased to 8.5 h (±1 h) at 96 mM adipic acid. This increase in lag phase was not observed in the osmotic control experiments (data not shown).

##### Effect of pH on Ethanol Red grown in the presence of adipic acid

When the pH of the medium was increased from pH 5 to pH 6, a number of differences were observed. During respiro-fermentation, cells were able to grow at higher concentrations of adipic acid, even at the maximum concentration of 650 mM. The negative effect on µ_max_ was milder at pH 6 than at pH 5: at 650 mM adipic acid, µ_max_ was reduced by 21% (±10%) at pH 6, compared with a reduction of 40% (±5%) at pH 5 in the presence already at 384 mM adipic acid (Fig. 3a).

During the respiratory phase, growth was detectable at pH 6 up to 192 mM adipic acid, whereas it was completely inhibited at pH 5. Although Ethanol Red cells could sustain higher concentrations of adipic acid at pH 6, µ_max_ was more affected than at pH 5. The error bars were also large at this pH (Fig. 3b). Furthermore, the lag phase between the respiro-fermentative and the respiratory growth phases was less affected at pH 6 than pH 5. Only at the highest concentration permitting growth (192 mM adipic acid) was a significant increase observed, from 4.2 h (±1.2 h) in the control with no adipic acid to 7.1 h (±0.3 h). In the osmotic control experiments no increase in the lag time was observed with increasing osmotic pressure (data not shown).

#### *Zygosaccharomyces bailii*

##### Effect of adipic acid on the growth of CBS 7555 at pH 5

**Fig. 4:**
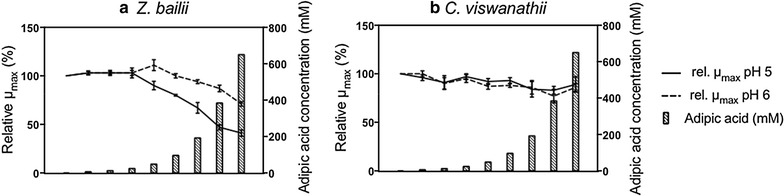
Effect of adipic acid on relative µ_max_ for *Z. bailii* (**a**) and *C. viswanathii* (**b**). The results are expressed as relative µ_max_, corrected for the effect of osmotic pressure, to the µ_max_ of cells grown without adipic acid. The concentrations of adipic acid are: 0, 6, 12, 24, 48, 96, 192, 384 and 650 mM. All results are given as the mean ± standard deviation from five replicates

Cells of *Z. bailii* were able to grow in the presence of all concentrations of adipic acid tested. At the highest concentration of 650 mM a long initial lag phase was detected, which was not observed in the osmotic control experiments (Additional file 1: Fig. S7). A significant reduction in µ_max_ was observed between adipic acid concentrations of 24 mM and 48 mM, and this decrease continued with increasing adipic acid concentration. At the highest concentration of adipic acid, the reduction in µ_max_ was 59% (±3%) (Fig. 4a), and the final OD before the diauxic shift was reduced by 43% (±7%), compared to the control with no adipic acid. Osmotic stress was not the cause of the observed growth inhibition (Additional file 1: Fig. S7).

After the diauxic shift, a fold change of only 0.7 (±0.1) was observed in the OD in the absence of adipic acid. This is due to the intrinsic metabolic features of *Z. bailii*, the respiro-fermentative metabolism of which is shifted more towards respiration than in the case of *S. cerevisiae* [35, 36]. The lag phase between the respiro-fermentative and the respiratory growth phases was affected by both adipic acid and osmotic pressure. For cells grown in presence of adipic acid the time for the lag phase increased with increasing adipic acid concentration, whereas no obvious trend in time for the lag phase with increasing osmotic pressure could be observed (Additional file 1: Fig. S7).

##### Effect of pH on CBS 7555 grown in the presence of adipic acid

When the pH of the medium was increased from pH 5 to pH 6, a number of differences were observed. There was no lag phase detected at 650 mM adipic acid, and µ_max_ decreased to a less extent than at pH 5 with increasing adipic acid concentration. At 650 mM, µ_max_ was reduced by 29% (±2%), while at pH 5 the reduction in the µ_max_ was 59% (±3%) (Fig. 4a). The lag phase between the respiro-fermentative and the respiratory growth phases was less affected by both adipic acid and osmotic pressure (Additional file 1: Fig. S7).

#### *Candida viswanathii*

##### Effect of adipic acid on the growth of NCYC 997

For neither pH 5 or pH 6 none of the adipic acid concentrations tested completely inhibited the growth of NCYC 997, and no lag phase could be detected at any concentration of adipic acid or in the osmotic control experiments (Additional file 1: Fig. S8). A slight decrease in µ_max_ was observed with increasing adipic acid concentration. In the presence of 650 mM adipic acid, the µ_max_ was reduced by 11% (±8%) at pH 5 and by 14% (±4) at pH 6 (Fig. 4b). After the diauxic shift, the OD increased by only 0.5 units, possibly suggesting that *C. viswanathii* has a primarily respiratory metabolism, as has been proposed for the closely related *Candida tropicalis* [37]. It should also be noted that cell shape may affect the OD values obtained with the Bioscreen [38]. It can, therefore, not be excluded that the increase in OD observed after the first growth phase was an artefact resulting from a change in cell shape over time, and not due to an increase in cell mass owing to respiration. The final OD was slightly affected by adipic acid and at 650 mM it was reduced by 24% (±2%), which was not observed in the osmotic control experiments. The decrease in final OD was lower at pH 6, and at 650 mM adipic acid it was reduced by 12% (±2%) (Additional file 1: Fig. S8).

##### Comparison of the tolerance of yeasts to adipic acid

All yeast species tested were able to grow under respiro-fermentative metabolism, or potentially full respiration as suggested for *C. viswanathii*, in the presence of adipic acid up to the maximum concentration of 650 mM, at both pH 5 and pH 6, apart from Ethanol Red, which did not grow at 650 mM adipic acid at pH 5. The maximum specific growth rate of all strains were less affected by adipic acid when grown at pH 6 compared to pH 5, apart from *C. viswanathii*, in which no difference could be observed. Among the yeasts investigated, *C. viswanathii* was the yeast with the highest tolerance to adipic acid at both pH 5 and pH 6 (Table 5).

**Table 5:** Reduction in µ_max_ during first growth phase of yeasts with 650 mM adipic acid

Species	Strain	pH 5 (%)	pH 6 (%)
*S. cerevisiae*	CEN.PK 113-7D	45 ± 4	24 ± 2
*S. cerevisiae*	Ethanol Red	n.d.^a^	30 ± 4
*Z. bailii*	CBS 7555	59 ± 3	29 ± 2
*C. viswanathii*	NCYC 977	11 ± 8	14 ± 4

The reduction in µ_max_ is corrected for osmotic pressure and compared to control with no adipic acid. Values given are the mean ± standard deviation of five replicates except for Ethanol Red where ten replicates were used

^a^No respiro-fermentative growth could be detected at 650 mM adipic acid at pH 5

##### Effect of adipic acid on the filamentous fungus *Aspergillus niger*

*Aspergillus niger* is a natural producer of citric acid and is used for industrial-scale production of citric acid at low pH [13]. Its ability to produce organic acids at low pH makes it an interesting candidate as a host for the production of adipic acid. In the present study, the tolerance of the wild type strain of *A. niger* (ATCC 1015) to adipic acid when grown on agar plates was evaluated.

##### Effect of adipic acid on the growth of *A. niger*

**Fig. 5:**
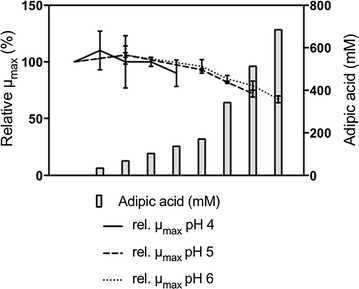
Effect of adipic acid on relative µ_max_ of *A. niger*. The results are expressed as relative µ_max_ to the µmax of cells grown without adipic acid. The concentrations of adipic acid for pH 5 and pH 6 are: 0, 68, 171, 342 and 513 mM. For pH 6 there is also 684 mM. For pH 4 the concentrations of adipic acid are: 0, 34, 68, 103 and 137 mM. All results are given as the mean ± standard deviation from three to six replicates

Growth was observed under all the conditions tested, and no significant difference in reduction of µ_max_ was seen between the three pH values (pH 4, pH 5 and pH 6) tested (Fig. 5). Compared to the control with no adipic acid, a significant reduction in µ_max_ was seen with increasing adipic acid concentration starting at >68 mM, at pH 5, showing a reduction of 28% (±3%) at 513 mM. At pH 6 a slight increase in µ_max_ was observed at 68 mM adipic acid, followed by a significant decrease in µ_max_ at adipic acid concentrations >171 mM, with a reduction in µ_max_ of 33% (±3%) at 684 mM. At pH 4, no significant difference in relative µ_max_ was observed at any adipic acid concentration tested (Fig. 5).

##### Effect of adipic acid on spore formation of *A. niger*

**Fig. 6:**
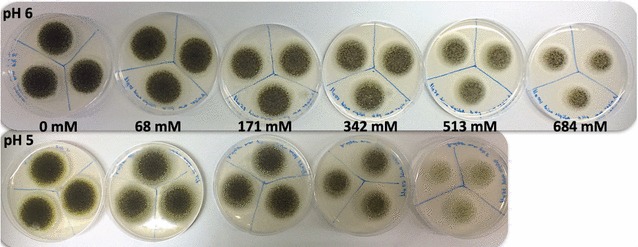
Effect of adipic acid and pH on spore formation of *A. niger*. Agar plates with triplicate cultures of *A. niger*, 5 days after inoculation at pH 6 (*above*) and pH 5 (*below*) at various adipic acid concentrations. Due to solubility issue there is no plate at 684 mM adipic acid for pH 5

From visual inspection of the colonies it was observed that spore formation was affected by adipic acid; less dense spore formation being seen with increasing adipic acid concentration at pH 5 and pH 6 (Fig. 6). At pH 4, no clear trend could be seen (data not shown). A difference in spore formation was seen depending on the pH, with less dense spores at pH 5 than at pH 6. This is clear when comparing the plates with 513 mM adipic acid concentration at pH 5 and 6.

### Comparison of the tolerance of all the microorganisms to adipic acid

Since the distribution between the various forms of adipic acid and hence, the amount of membrane-diffusible adipic acid, varies with pH, the comparison of all the microorganisms investigated in this study must be made by considering cultures grown at the same pH. All the microorganisms included in this study were able to grow in the control cultures with no adipic acid at pH 6, and were therefore compared at this pH.

#### Yeasts and *A. niger* tolerate far higher titres of adipic acid than bacteria

**Fig. 7:**
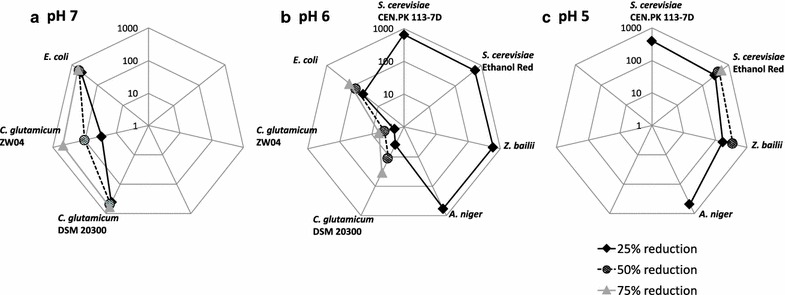
Comparing the effect of adipic acid and pH on the relative µ_max_ for all the microorganisms included in this study. Data points are estimated concentration of adipic acid required to reduce µ_max_ by 25, 50 and 75% for bacteria at pH 7 (**a**), yeasts, *A. niger* and bacteria at pH 6 (**b**), and yeasts and *A. niger* at pH 5 (**c**). The axes on the radar plots are logarithmic base 10. *C. viswanathii* is not included since a 25% reduction in µ_max_ was not observed at any adipic acid concentration at any pH tested. The values given are obtained from five to ten replicates

The maximum specific growth rate was reduced by 25% already at very low concentrations of adipic acid (e.g., <10 mM for the *C. glutamicum* strains and ~50 mM for *E. coli*). A similar reduction in µ_max_ was not observed in any yeast species or *A. niger* until higher concentrations of adipic acid (as high as >550 mM) (Fig. 7). For the yeast *C. viswanathii* a 25% reduction in µ_max_ was not observed at any of the adipic acid concentrations tested. This comparison, at 25% reduction in µ_max_, indicates that yeasts tolerate roughly 10–100 times higher concentrations of adipic acid than bacteria. A 50% reduction in µ_max_ was not observed in any yeast or *A. niger*, whereas a 75% reduction was observed in all three bacterial strains, even at adipic acid concentrations below 150 mM.

#### Bacteria are more affected by the degree of dissociation of adipic acid than yeasts and *A. niger*

All the microorganisms included in this study were more affected by adipic acid at lower pH, in other words, in the presence of a greater fraction of undissociated adipic acid, than at higher pH (less undissociated acid). The amount of undissociated adipic acid increased from negligible amounts at pH 7–0.5% at pH 6. Although the difference in the amount of undissociated adipic acid was low, roughly 10–100 times higher total adipic acid concentration was required to reduce µ_max_ of bacteria by 25% at pH 7, compared to pH 6. When the pH was further decreased from pH 6 to pH 5, the amount of undissociated adipic acid increased from 0.5 to 13.8%. Although the increase in undissociated adipic acid was greater for yeasts and *A. niger* compared with bacteria, only 1–4 times higher total adipic acid concentration was required to reduce µ_max_ by 25% at pH 6 compared to pH 5. Therefore, it can be concluded that bacteria are more affected by the presence of diffusible undissociated adipic acid than yeasts and *A. niger.*

## Discussion

Due to the high global demand for adipic acid and the unsustainable petroleum based process currently used, biobased-production of adipic acid is one of the primary targets for platform chemicals in biorefineries. Establishing a microbial cell factory for adipic acid production from renewable sources is one of the options that can be considered [8, 9]. Tolerance to adipic acid is a critical factor that should be considered when selecting a suitable host for biobased-production; however, to the best of our knowledge, no studies addressing this matter have yet been published. In the present study, we investigated the levels of tolerance of a range of microorganisms to adipic acid, from yeasts filamentous fungi and bacteria.

In the following sections the mode of entry of adipic acid is hypothesized to be mainly via passive diffusion of the undissociated form of adipic acid [18, 19]. It should be emphasized that in this study neither extra- nor intracellular concentrations of adipic acid was determined; therefore the following discussion aims to put forth hypotheses based on the observed differences in growth between the studied microorganisms in the presence of adipic acid. Hence, further experiments will be necessary to prove the actual mode of inflow and action of adipic acid on the cellular physiology leading to the effect we observed on the specific growth rate.

### Why do yeasts and *A. niger* have greater tolerance to adipic acid than bacteria?

When comparing the tolerance levels exhibited by the investigated microorganisms at the same pH, it was clear that yeasts and *A. niger* tolerated substantially higher concentrations of adipic acid than bacteria. There may be several reasons for these differences associated with fundamental physiological differences between these microorganisms. These differences are addressed and discussed below in terms of their potential influence in determining the level of tolerance to adipic acid.

The surface-to-volume ratio may affect the tolerance to adipic acid. This ratio increases with decreasing cell size, and will thus be greater for bacteria than for yeasts and *A. niger*. More acid can diffuse into smaller cells (i.e., bacteria), which may explain the better tolerance of yeasts and *A. niger* to the acid.

The membrane composition may also affect the rate of diffusion of the undissociated form of adipic acid through the membrane. Sphingolipids, which have been found to be present in the cell membranes of yeasts, have been shown to be important for tolerance to both acids [36, 39] and low pH [40]. Sterols, present in the membrane of eukaryotes but only found to be present in very few bacteria, are important in controlling the cell permeability [41]. Since there are no sphingolipids in bacterial cell membranes and sterols are not commonly found [41, 42], bacterial membranes are probably more permeable to undissociated adipic acid. Therefore, extracellular undissociated adipic acid can cross bacterial membranes at a higher rate than yeast cell membranes, thus causing higher detoxification demands. A potentially higher diffusion rate of the extracellular undissociated adipic acid will also increase the shift of the extracellular equilibrium between the different adipic acid forms towards the undissociated one, which then can diffuse over the membrane and continue to cause higher detoxification demands.

An additional factor potentially influencing the level of tolerance to adipic acid is associated with the presence of transporters for the active transport of specific molecules, possibly including the dissociated forms of adipic acid. No specific membrane transporters for adipic acid have been reported so far, although the citrate transporter CitP of *Lactococcus lactis* has been shown to have very broad substrate specificity including the ability to transport adipic acid intracellularly [43]. Despite of the lack of knowledge on specific adipic acid transporters, it could be hypothesized that adipic acid may be transported by other di- or tri-carboxylic acid transporters. In fact, these transporters have been reported in both yeast and bacteria [44–48]. Based on the studies published, it is difficult to conclude whether there are any relevant differences between the transporters in yeasts and bacteria that could explain the higher tolerance of yeasts to adipic acid. However, altering the substrate specificity of organic acid transporters or knocking out specific transporters of adipic acid could provide a strategy for increasing the tolerance to adipic acid.

Cell compartmentalization, which is typical in eukaryotic systems such as yeasts and filamentous fungi, may also play a role in determining the observed difference in tolerance. In bacteria, all macromolecules are found unprotected in the cytosol where also all the metabolic reactions take place. Using the tricarboxylic acid cycle (TCA cycle) as an example, any acid molecule able to inhibit the TCA cycle, entering a bacterial cell can directly interfere with the enzymes of the TCA cycle, since the TCA cycle takes place directly in the cytosol. In contrast, in order to exert an inhibitory effect on the TCA cycle in eukaryotic systems, the acid molecule must also pass through the mitochondrial membrane. Furthermore, the near-neutral pH of the cytosol leads to acid deprotonation, thereby hindering its further potential diffusion through the mitochondrial membrane. In addition, eukaryotic systems contain vacuoles which play a role in the homeostasis of several ions and also of the intracellular pH [49]. The vacuole lumen has been shown to have an acidic pH due to the action of vacuolar membrane ATPases. The acidic pH of the vacuole is also crucial for cellular functionality, as the proton gradient thus generated is used for other essential transport mechanisms across the vacuolar membrane [50]. The presence of vacuoles thus provides eukaryotes with a means of decreasing the concentration of cytosolic protons and in the case of adipic acid provides higher tolerance to protons from undissociated adipic acid.

The cytosolic pH, which affects the form of adipic acid once it is in the intracellular environment, may also play a role in acid tolerance. The cytosolic pH of bacteria and yeasts included in this study is at optimal growth conditions approximately 7.5 [15, 51, 52] and 7.0 [53] respectively. The amount of fully dissociated adipic acid at pH 7.0 and 7.5 is 97.5 and 99.2%, respectively, resulting in slightly more of the protons released from undissociated adipic acid entering in bacteria than in yeast. Due to this difference, the concentration gradient, and hence the diffusion rate of undissociated adipic acid, is slightly higher in bacteria than yeast. The difference in pH means that the internal concentration of protons in yeasts is roughly three times higher than that in bacteria and thus, yeasts may be better adapted to acidic environments than bacteria. This could be one reason why yeasts can tolerate lower pH than bacteria and are better adapted to acid stress.

Under optimal conditions, ATP is generated for cell formation and maintenance. When cells encounter acid stress, there is an additional need for ATP by the cellular H^+^ pumps to remove H^+^ ions from the cytosol to maintain the intracellular pH at physiological values [53, 54]. This additional need for ATP re-directs the ATP formed away from cell mass formation and possibly also maintenance. ATP is generated via respiration, by the complete oxidation of glucose to CO_2_, and/or via substrate-level phosphorylation (i.e. glycolysis), generating less oxidized products, such as ethanol in the case of *S. cerevisiae* and acetic acid in the case of *E. coli*. In some microorganisms ATP can be generated via a combination of respiration and substrate-level phosphorylation. In terms of the ATP generated per glucose molecule, respiration yields more ATP than substrate-level phosphorylation. As a result of the difference in ATP generation, microorganisms using respiration are more likely to generate enough ATP to cope with acid stress (assuming otherwise similar conditions).

The yeasts *S. cerevisiae* and *Z. bailii* are both Crabtree-positive and ATP is formed via both respiration and substrate-level phosphorylation. In the case of *C. viswanathii*, on the other hand, the predominant path of ATP generation is likely to be respiration [37]. The fact that µ_max_ was reduced by roughly 30% when *C. viswanathii* was cultivated in a microplate, compared to a shake flask (data not shown) further supports this theory, since it is known that the microplate cultivations used in the present study result in slight oxygen limitation [31]. As there is no way to direct ATP production towards substrate-level phosphorylation, the rate of ATP production will probably decrease with decreasing oxygen availability.

*Escherichia coli* is able to respiro-ferment via mixed acid fermentation, generating ATP via the formation of acetic acid. However, if oxygen is available, respiration is preferred over substrate-level phosphorylation [55]. *C. glutamicum* is also able to use mixed acid fermentation for the metabolism of glucose, but under such conditions cell growth is arrested [56]. In the present study, µ_max_ was decreased by roughly 50% in both *C. glutamicum* strains when cultivated in microplates compared to shake flasks (data not shown). This may indicate that the metabolism and hence the route for ATP formation shifted towards a higher flux via substrate-level phosphorylation, and hence less ATP was formed when cells were cultivated under slight oxygen limitation in the microplate. Although both *S. cerevisiae* and *Z. bailii* prefer the less ATP-yielding substrate-level phosphorylation over respiration, these strains were less affected by adipic acid, in terms of both relative µ_max_ and final OD when grown on glucose, compared to bacteria, indicating that ATP generation is not the main reason for the higher tolerance of the yeasts *S cerevisiae* and *Z. bailii* to adipic acid than that exhibited by bacteria. The inflow of adipic acid (by passive diffusion or other means) is probably higher in bacteria than yeasts, resulting in a greater reduction in µ_max_ and less cell mass being formed. The observation that bacteria were more affected by an increase in undissociated adipic acid than yeasts and *A. niger* further supports the hypothesis of a higher inflow of adipic acid in bacteria than in yeasts and *A. niger*.

When the metabolism of *S. cerevisiae* changed from respiro-fermentation to full respiration the lag phase between the two growth phases increased with increasing adipic acid concentration. During this lag phase, as glucose is depleted, stress responses are induced, and gene expression is reprogrammed such that the genes controlling fermentative metabolism are down-regulated and those controlling respiratory metabolism are upregulated [57, 58]. In addition to the stress to which the cells are exposed due to glucose starvation, cells cultivated in the presence of adipic acid are subjected to an additional, acid, stress, requiring detoxification. This requires ATP, probably diverting ATP from reprogramming of the metabolism, thus leading to an extension of the lag phase. After the diauxic shift, µ_max_ of *S. cerevisiae* was strongly affected by increasing adipic acid concentration. Assuming there are no major changes in membrane composition or the transporters present, the inflow of adipic acid should be unaffected. The interference of enzymes in the TCA cycle by adipic acid may explain why respiration was more affected, despite the higher ATP yield. These enzymes have specificity for diacids, including succinic acid, which is two carbons shorter than adipic acid. The dicarboxylic acid, malonic acid, has previously been reported to inhibit oxidation of both succinate and oxaloacetate in the TCA cycle [59]. The hypothesis that adipic acid interferes with the TCA cycle would also provide an additional explanation of why bacteria were more affected than yeasts and *A. niger*, since the respiratory metabolism in bacteria takes place in the inner membrane and is therefore unprotected, compared to yeast and *A. niger*, where it takes place inside the mitochondria.

As discussed above, there are several differences between yeasts, *A. niger* and bacteria, in terms of their overall physiology and their paths of ATP generation. From the results obtained in this study it cannot be concluded which of these factors has the greatest effect on tolerance to adipic, and the differences in tolerance are most likely due to a combination of all the physiological and metabolic differences discussed above.

### Adipic acid tolerance in solid versus liquid media

Due to limitations inherent to the instrument used to monitor biomass growth, the methods for screening the effect of adipic acid on microbial growth differed between yeast and bacteria, which were studied in liquid media, compared with the filamentous fungus *A. niger* which was studied using a solid agar-media. As different methodologies was applied the level of tolerance to adipic acid can not be directly compared. Nevertheless, we believe that the results of the present study are representative of the level of tolerance of *A. niger* to adipic acid. Further studies of *A. niger* submerged cultures will be necessary to better quantify the level of tolerance of *A. niger* to adipic acid.

### Lysine overproduction may affect tolerance to adipic acid

**Fig. 8:**
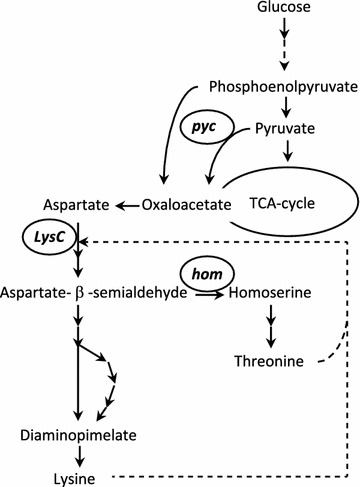
Pathway and primary regulation of l-lysine biosynthesis in *Corynebacterium glutamicum*, modified from [60]. The *broken arrow* indicates feed-back inhibition. The genes encoding the enzymes mutated in the ZW04 strain are circled and named as follows: *pyc* pyruvate carboxylase, *lysC* aspartokinase, *hom* homoserine dehydrogenase

The lysine overproducing strain, *C. glutamicum* ZW04, was less tolerant to adipic acid at both pH 6 and pH 7, than its parental wild type strain *C. glutamicum* DSM 20300. The difference between these two strains lies in three mutations in the lysine-producing pathway, namely: *lysC* (T311I), *hom* (V59A) and *pycA* (P458S) [60]. These modifications increase the flux towards lysine at the expense of the TCA flux [29, 60] (Fig. 8). In addition, the production of lysine requires NADPH, which can be balanced by an increased flux through the pentose phosphate pathway [61]. The reduced flux in the TCA cycle, together with an increase in flux through the pentose phosphate pathway, is likely to lead to the generation of less ATP and, consequently, this strain would be less efficient than the wild type in reactions requiring ATP such as pumping out adipate and protons.

### Suitable host for future adipic acid production

In order for the biobased production of adipic acid to be economically feasible, the microbial host must tolerate acid titres of 50–100 g/L adipic acid [14, 15] (corresponding to 340–680 mM). At these adipic acid concentrations, all bacteria included in this study lost their ability to grow, even at pH 7 and therefore, they appear not to be suitable candidates for biobased adipic acid production. However, if the titres could be reduced, and the process run at neutral pH, without having too severe impact on the economy of the process, a bacterial host could serve as a possible adipic acid producer.

*Aspergillus niger* and all yeasts investigated, apart from Ethanol Red, were able to grow on glucose in presence of 384 mM and 650 mM adipic acid at pH 5. *C. viswanathii* was the yeast species least affected by adipic acid, and its growth was almost unaffected, even at the highest adipic acid concentration (650 mM), also at pH 5. Tolerance to adipic acid at low pH is an additional advantage due to the reduced cost of downstream processing [16]. Plausible explanations why *C. viswanathii* is more tolerant to adipic acid than the other yeast species included in this study may be associated with membrane composition and functionality. This species has been reported to grow on fatty acids and alkanes as sole carbon source [7, 33].

*Candida viswanathii* may thus have evolved a membrane with low permeability for fatty acids, which could also mean it is less permeable to adipic acid, and hence more tolerant. Engineering *C. viswanathii* to a host that can produce adipic acid requires knowledge about its genome, and a molecular toolbox to make the necessary genetic alterations. Although *C. viswanathii* has been much less studied than, for instance, the model yeast *S. cerevisiae*, its genome is available [62], and there are several reports on genetic modifications indicating molecular tools are available [63–66]. However, it may be suggested that *C. viswanathii* has primarily respiratory metabolism, as has been proposed for the closely related *Candida tropicalis* [37], thus anaerobic fermentation cannot be utilized, and the overall cost of supplying the reactor with oxygen will increase. This cost could be avoided by using the well characterized yeast *S. cerevisiae* as a potential host, since it is able to ferment under anaerobic conditions, and showed high tolerance to adipic acid, although the industrial strain Ethanol Red was sensitive to adipic acid at concentrations of 384 mM and above.

## Conclusions

In this screening study, yeasts and *A. niger* were found to have substantially higher tolerance to the total adipic acid concentration than the investigated bacterial strains. Yeasts and *A. niger* were also found to tolerate a greater increase in the membrane-diffusible form of undissociated adipic acid than bacteria, due to a decrease in the pH. The yeast species *C. viswanathii* exhibited the highest tolerance to adipic acid and was almost unaffected by the presence of even the highest adipic acid concentration tested (650 mM). Changing the pH had no effect on *C. viswanathi* level of tolerance, which is beneficial in an industrial setting due to the reduced cost of downstream processing at lower pH.

Possible reasons why bacteria were more affected by adipic acid than yeasts and *A. niger* were discussed. Overall, there are several plausible explanations including differences in surface-to-volume ratio, membrane composition, membrane transporters, cytosolic pH, cell compartmentalization and ATP generation. The difference observed in the tolerance of bacteria and yeasts is probably not due to only one of these parameters, but rather a combination of them.

It was also noted that respiratory growth of *S. cerevisiae* was more affected than respiro-fermentative growth with increasing adipic acid concentration. It was suggested that the reason for this could be inhibition of the enzymes in the TCA cycle by adipic acid.

## Additional file

**Additional file 1.** Growth curves from microtiter plate cultivations. The data contains growth curves for all microorganisms include in the study cultivated in microtiter plates.
