# Supplementary material for: Adipic acid tolerance screening for potential adipic acid production hosts
Source: Microb Cell Fact. 2017 Feb 1;16:20. doi: 10.1186/s12934-017-0636-6 (PMC5286774; doi:10.1186/s12934-017-0636-6)
Supplement: Supplementary file 1 — Additional file 1. Growth curves from microtiter plate cultivations. The data contains growth curves for all microorganisms include in the study cultivated in microtiter plates. [file 12934_2017_636_MOESM1_ESM.docx]

Supporting information for

**Adipic acid tolerance screening for potential adipic acid production hosts**

Emma Karlsson ([Emma.Karlsson@chalmers.se](mailto:Emma.Karlsson@chalmers.se))^1^, Valeria Mapelli ([Valeria.Mapelli@chalmers.se](mailto:Valeria.Mapelli@chalmers.se))^1,2^, Lisbeth Olsson ([Lisbeth.Olsson@chalmers.se](mailto:Lisbeth.Olsson@chalmers.se))^1^*

*corresponding author

^1^Department of Biology and Biological Engineering, Division of Industrial Biotechnology, Chalmers University of Technology, Gothenburg, Sweden

^2^Sacco S.r.l., Cadorago (CO), Italy

**Figure S1-S8** contains growth curves visualized as OD_corrected_ from all microplate cultivations performed in the study.

Fig. S1

Growth curves for Corynebacterium glutamicum DSM 20300 at pH 6 and pH 7 in the presence of adipic acid and “osmotic control” where cultures are subjected to the same osmotic pressure (by addition of KCl) as the one resulting from adipic acid addition. Each growth curve is an average of 5 replicates. In the osmotic test, for each condition the corresponding concentration of adipic acid is reported

Fig. S2

Growth curves for Corynebacterium glutamicum ZW04 at pH 6 and pH 7 in the presence of adipic acid and “osmotic control” where cultures are subjected to the same osmotic pressure (by addition of KCl) as the one resulting from adipic acid addition. Each growth curve is an average of 5 replicates. In the osmotic test, for each condition the corresponding concentration of adipic acid is reported

Fig. S3

Growth curves for Escherichia coli K12 MG1655 at pH 6 and pH 7 in the presence of adipic acid and “osmotic control” where cultures are subjected to the same osmotic pressure (by addition of KCl) as the one resulting from adipic acid addition. Each growth curve is an average of 5 replicates. In the osmotic test, for each condition the corresponding concentration of adipic acid is reported

Fig. S4

Growth curves for Saccharomyces cerevisiae CEN.PK 113-7D at pH 5 and pH 6 in the presence of adipic acid and “osmotic control” where cultures are subjected to the same osmotic pressure (by addition of KCl) as the one resulting from adipic acid addition. Each growth curve is an average of 5 replicates. In the osmotic test, for each condition the corresponding concentration of adipic acid is reported

Fig. S5

Growth curves for Saccharomyces cerevisiae Ethanol Red at pH 5 and pH 6 in the presence of adipic acid and “osmotic control” where cultures are subjected to the same osmotic pressure (by addition of KCl) as the one resulting from adipic acid addition. Each growth curve is an average of 5 replicates. In the osmotic test, for each condition the corresponding concentration of adipic acid is reported

Fig. S6

Growth curves for Saccharomyces cerevisiae Ethanol Red at pH 5 and pH 6 in the presence of adipic acid and “osmotic control” where cultures are subjected to the same osmotic pressure (by addition of KCl) as the one resulting from adipic acid addition. Each growth curve is an average of 5 replicates. In the osmotic test, for each condition the corresponding concentration of adipic acid is reported

Fig. S7

Growth curves for Zygosaccharomyces bailii at pH 5 and pH 6 in the presence of adipic acid and “osmotic control” where cultures are subjected to the same osmotic pressure (by addition of KCl) as the one resulting from adipic acid addition. Each growth curve is an average of 5 replicates. In the osmotic test, for each condition the corresponding concentration of adipic acid is reported

Fig. S8

Growth curves for Candida viswanathii at pH 5 and pH 6 in the presence of adipic acid and “osmotic control” where cultures are subjected to the same osmotic pressure (by addition of KCl) as the one resulting from adipic acid addition. Each growth curve is an average of 5 replicates. In the osmotic test, for each condition the corresponding concentration of adipic acid is reported
